# Supplementary material for: COVID-19 Vaccine Mandates: Attitudes and Effects on Holdouts in a Large Australian University Population
Source: Int J Environ Res Public Health. 2022 Aug 16;19(16):10130. doi: 10.3390/ijerph191610130 (PMC9408755; doi:10.3390/ijerph191610130)
Supplement: Supplementary file 1 [file ijerph-19-10130-s001.zip › ijerph-1807366-supplementary.pdf]

Background

Which best describes your primary role at UWA?

Academic staff

Professional staff

Undergraduate student

Postgraduate student

Other (please indicate below)

At which Campus are you mainly based:

Crawley (main)

Nedlands

Queen Elizabeth II Medical Centre

Albany

Other

What is your age?

Under 18

18 - 24

25 - 34

35 - 44

45 - 54

55 - 64

65 - 74

75 - 84

85 or older

Prefer not to say

What gender do you identify as?

Male

Female

Non-binary / third gender

Prefer not to say

Do you have a health condition that makes you more susceptible to COVID-19 (such as immune comprising conditions or treatments, as well as pregnancy)?

No

Yes

If you are happy to to provide this information, please share the health condition that makes you more susceptible to COVID-19 (otherwise leave blank)

Vaccination status

Regarding your COVID-19 vaccination status, the following questions are about Therapeutic Goods Administration (TGA) approved COVID-19 vaccines.

Please indicate which of the following is true - I have/am:

a) Received both first and second doses of a COVID-19 vaccine

b) Received the first dose of a COVID-19 vaccine and plan to get a second dose

c) Received the first dose but do not plan to receive any more doses

d) Booked or plan to receive the first dose of a COVID-19 vaccine

e) Obtained formal medical exemption from COVID-19 vaccination via the Australian Immunisation Register (AIR)

f) Not intending to get vaccinated against COVID-19 for my own reasons

g) Undecided about whether to be vaccinated against COVID-19

h) I opt to not disclose my vaccination status

Regarding booster shots, I am:

a) Willing to get booster shots

b) Not willing to get booster shots

c) Not sure whether I am willing to get booster shots

What are your main reasons for this decision?

Mandate perception

Would you support a mandate by the WA Government that requires everyone attending campus to be vaccinated, if they are able to be?

Strongly oppose | Somewhat oppose | Neutral | Somewhat support | Strongly support

What are the main reasons for your position?

In the absence of a government mandate, would you support a mandate by the University that requires everyone attending campus to be vaccinated, if they are able to be?

Strongly oppose | Somewhat oppose | Neutral | Somewhat support | Strongly support

What are the main reasons for your position?

Factors impacting vaccine likelihood

How would each of the following impact the likelihood that you will get vaccinated for COVID-19:

	Decrease likelihood	No change in likelihood	Increase likelihood
An information session about COVID-19 vaccines with scientific experts	○	○	○
International border opening	○	○	○
State border opening	○	○	○
A mandate policy where job loss or exclusion from study is a consequence of refusing COVID-19 vaccination	○	○	○

Vaccine beliefs

The following questions are about your beliefs relating to COVID-19 vaccinations on campus.

To what extent do you agree/disagree with the following statements:

a) "I feel safer coming to campus if others are vaccinated"

Strongly disagree | Somewhat disagree | Neither agree nor disagree | Somewhat agree | Strongly agree

b) "People should be able to attend campus even if they choose not to vaccinate"

Strongly disagree | Somewhat disagree | Neither agree nor disagree | Somewhat agree | Strongly agree

c) "I believe COVID vaccines are safe, effective and necessary"

Strongly disagree | Somewhat disagree | Neither agree nor disagree | Somewhat agree | Strongly agree

d) "The government should mandate COVID-19 vaccinations for the wider community to work, travel, or attend events / hospitality venues"

Strongly disagree | Somewhat disagree | Neither agree nor disagree | Somewhat agree | Strongly agree

e) "People should be exempt from vaccine mandates for religious or personal belief reasons"

Strongly disagree | Somewhat disagree | Neither agree nor disagree | Somewhat agree | Strongly agree

f) "UWA staff and students who refuse to vaccinate should lose their positions."

Strongly disagree | Somewhat disagree | Neither agree nor disagree | Somewhat agree | Strongly agree

g) "Each individual has a duty to vaccinate to protect those we work or study with"

Strongly disagree | Somewhat disagree | Neither agree nor disagree | Somewhat agree | Strongly agree

Further comments

Do you have any further comments about the University potentially mandating COVID- 19 vaccinations?
