# Supplementary material for: COVID-19 Vaccine Mandates: Attitudes and Effects on Holdouts in a Large Australian University Population
Source: Int J Environ Res Public Health. 2022 Aug 16;19(16):10130. doi: 10.3390/ijerph191610130 (PMC9408755; doi:10.3390/ijerph191610130)
Supplement: Supplementary file 1 [file ijerph-19-10130-s001.zip › Supplementary Table S1 Participant demographics.pdf]

Supplementary Table S1. Participant demographics.

Gender	
**Female**	56.81%
**Male**	40.62%
**Non-binary**	1.15%
**Prefer not to say**	1.42%

Age	
**18 - 24**	40.24%
**25 - 34**	18.80%
**35 - 44**	15.46%
**45 - 54**	12.06%
**55 - 64**	9.03%
**65 - 74**	2.95%
**75 - 84**	0.28%
**85 or older**	0.14%
**Prefer not to say**	0.69%
**Under 18**	0.35%

Occupation	
**Undergraduate**	35.79%
**Postgraduate**	28.04%
**Professional staff**	19.63%
**Academic staff**	13.62%
**Other**	2.92%

Underlying Health Condition	
**No**	91.00%
**Yes**	9.00%
