# Supplementary material for: COVID-19 Vaccine Mandates: Attitudes and Effects on Holdouts in a Large Australian University Population
Source: Int J Environ Res Public Health. 2022 Aug 16;19(16):10130. doi: 10.3390/ijerph191610130 (PMC9408755; doi:10.3390/ijerph191610130)
Supplement: Supplementary file 1 [file ijerph-19-10130-s001.zip › Supplementary Table S2 Comorbidity List.pdf]

**Supplementary Table S2: Comorbidities self-identified by participants**						
Type 1 Diabetes	Immune suppressant medication	Thyroidectomy following acute autoimmune thyroiditis, primary parahyperthryoidis, severe asthma.	Sarcoidosis	Myasthenia gravis	MCTD	In recovery from thyroid cancer
Pregnancy	Underactive thyroid function	Susceptible to bronchitis. Have had pneumonia but not hospitalised. Just tend to get coughs!	Respiratory illness, weak immune response to respiratory diseases.	myalgic encephalomyelitis	Mannose-Binding-Lectin (MBL) deficiency	Immunocompromised (liver transplant)
Rheumatoid Arthritis (+ on immunomodulators)	Ulcerative colitis	Stomach issues and lowered immunity	Pulmonary stenosis	My biggest health condition makes me susceptible to infections which puts extra pressure on my immune system, in turn making me more likely to contract viruses	Lymphoedema after breast cancer	Immune suppressant medication for Crohn's disease treatment.
Psoriatic arthritis	Ulcerative colitis and endometriosis	Crohn’s disease	Pneumonia in both lungs as a child. Asthma and anaphylaxis which is an issue considering the COVID vaccines (which can make me more susceptible) plus immune system issues.	Moderate idiopathic hypertension, controlled by 40mg Telmisartan, 10mg Amlodipine once daily	Lung scarring from childhood whooping cough and pneumonia	IgA deficiency
Lupus	High blood pressure/allergies	Coeliac	Overweight/Obesity	Mitral valve prolapse	Juvenile Rheumatoid Arthritis – on immunosuppressant treatments.	
