# Supplementary material for: COVID-19 Vaccine Mandates: Attitudes and Effects on Holdouts in a Large Australian University Population
Source: Int J Environ Res Public Health. 2022 Aug 16;19(16):10130. doi: 10.3390/ijerph191610130 (PMC9408755; doi:10.3390/ijerph191610130)
Supplement: Supplementary file 1 [file ijerph-19-10130-s001.zip › Supplementary Tables S3-6 Vax Status Demographic.pdf]

Supplementary Materials Tables S3-6.

Participants' Vaccination Status (Double Vaccinated or Willing To Be, DVWTB) by Demographic Variables

Table S3. Vaccination Status as a function of Gender

Contingency Tables

Gender		Vaccination Status (DVWTB)		
		No	Yes	Total
Female	Observed	99	1536	1635
	% within row	6.1 %	93.9 %	100.0 %
Male	Observed	96	1073	1169
	% within row	8.2 %	91.8 %	100.0 %
Non-binary / third gender	Observed	3	30	33
	% within row	9.1 %	90.9 %	100.0 %
Prefer not to say	Observed	18	23	41
	% within row	43.9 %	56.1 %	100.0 %
Total	Observed	216	2662	2878
	% within row	7.5 %	92.5 %	100.0 %

Table S4. Vaccination Status as a function of Age

Contingency Tables

Age		Vaccination Status (DVWTB)		
		No	Yes	Total
18 - 24	Observed	99	1059	1158
	% within row	8.5 %	91.5 %	100.0 %
25 - 34	Observed	38	503	541
	% within row	7.0 %	93.0 %	100.0 %
35 - 44	Observed	35	410	445
	% within row	7.9 %	92.1 %	100.0 %
45 - 54	Observed	19	328	347
	% within row	5.5 %	94.5 %	100.0 %
55 - 64	Observed	12	248	260
	% within row	4.6 %	95.4 %	100.0 %
65 - 74	Observed	0	85	85
	% within row	0.0 %	100.0 %	100.0 %
75 - 84	Observed	0	8	8
	% within row	0.0 %	100.0 %	100.0 %
85 or older	Observed	0	4	4
	% within row	0.0 %	100.0 %	100.0 %
Prefer not to say	Observed	11	9	20
	% within row	55.0 %	45.0 %	100.0 %
Under 18	Observed	2	8	10
	% within row	20.0 %	80.0 %	100.0 %
Total	Observed	216	2662	2878
	% within row	7.5 %	92.5 %	100.0 %

Table S5. Vaccination Status as a function of Occupation

Contingency Tables

Occupation		Vaccination Status (DVWTB)		Total
		No	Yes	
Academic staff	Observed	6	386	392
	% within row	1.5 %	98.5 %	100.0 %
Other (please indicate below)	Observed	22	62	84
	% within row	26.2 %	73.8 %	100.0 %
Postgraduate student	Observed	66	741	807
	% within row	8.2 %	91.8 %	100.0 %
Professional staff	Observed	30	535	565
	% within row	5.3 %	94.7 %	100.0 %
Undergraduate student	Observed	92	938	1030
	% within row	8.9 %	91.1 %	100.0 %
Total	Observed	216	2662	2878
	% within row	7.5 %	92.5 %	100.0 %

Table S6. Vaccination Status as a function of Health Status

Contingency Tables

Underlying Health Condition		Vaccination Status (DVWTB) No	Yes	Total
No	Observed	194	2425	2619
	% within row	7.4 %	92.6 %	100.0 %
Yes	Observed	22	237	259
	% within row	8.5 %	91.5 %	100.0 %
Total	Observed	216	2662	2878
	% within row	7.5 %	92.5 %	100.0 %
