# Supplementary material for: COVID-19 Vaccine Mandates: Attitudes and Effects on Holdouts in a Large Australian University Population
Source: Int J Environ Res Public Health. 2022 Aug 16;19(16):10130. doi: 10.3390/ijerph191610130 (PMC9408755; doi:10.3390/ijerph191610130)
Supplement: Supplementary file 1 [file ijerph-19-10130-s001.zip › Supplementary Tables S7-10 Uni Mand Stance Demographic.pdf]

Supplementary Materials Tables S7-10.

Participants' Vaccination Status (Double Vaccinated or Willing To Be, DVWTB) by Demographic Variables

Table S7. Vaccination Status as a function of Gender

Contingency Tables

Gender		University Vaccine Mandate Stance			
		Neutral	Oppose	Support	Total
Female	Observed	85	196	1354	1635
	% within row	5.2 %	12.0 %	82.8 %	100.0 %
Male	Observed	68	213	888	1169
	% within row	5.8 %	18.2 %	76.0 %	100.0 %
Non-binary / third gender	Observed	0	4	29	33
	% within row	0.0 %	12.1 %	87.9 %	100.0 %
Prefer not to say	Observed	2	25	14	41
	% within row	4.9 %	61.0 %	34.1 %	100.0 %
Total	Observed	155	438	2285	2878
	% within row	5.4 %	15.2 %	79.4 %	100.0 %

Table S8. Vaccination Status as a function of Age

Contingency Tables

Age		University Vaccine Mandate Stance			
		Neutral	Oppose	Support	Total
18 - 24	Observed	67	199	892	1158
	% within row	5.8 %	17.2 %	77.0 %	100.0 %
25 - 34	Observed	35	89	417	541
	% within row	6.5 %	16.5 %	77.1 %	100.0 %
35 - 44	Observed	24	71	350	445
	% within row	5.4 %	16.0 %	78.7 %	100.0 %
45 - 54	Observed	16	38	293	347
	% within row	4.6 %	11.0 %	84.4 %	100.0 %
55 - 64	Observed	10	24	226	260
	% within row	3.8 %	9.2 %	86.9 %	100.0 %
65 - 74	Observed	2	1	82	85
	% within row	2.4 %	1.2 %	96.5 %	100.0 %
75 - 84	Observed	0	0	8	8
	% within row	0.0 %	0.0 %	100.0 %	100.0 %
85 or older	Observed	0	0	4	4
	% within row	0.0 %	0.0 %	100.0 %	100.0 %
Prefer not to say	Observed	0	12	8	20
	% within row	0.0 %	60.0 %	40.0 %	100.0 %
Under 18	Observed	1	4	5	10
	% within row	10.0 %	40.0 %	50.0 %	100.0 %
Total	Observed	155	438	2285	2878
	% within row	5.4 %	15.2 %	79.4 %	100.0 %

Table S9. Vaccination Status as a function of Occupation

Contingency Tables

Occupation		University Vaccine Mandate Stance			Total
		Neutral	Oppose	Support	
Academic staff	Observed	16	35	341	392
	% within row	4.1 %	8.9 %	87.0 %	100.0 %
Other (please indicate below)	Observed	2	29	53	84
	% within row	2.4 %	34.5 %	63.1 %	100.0 %
Postgraduate student	Observed	50	128	629	807
	% within row	6.2 %	15.9 %	77.9 %	100.0 %
Professional staff	Observed	33	60	472	565
	% within row	5.8 %	10.6 %	83.5 %	100.0 %
Undergraduate student	Observed	54	186	790	1030
	% within row	5.2 %	18.1 %	76.7 %	100.0 %
Total	Observed	155	438	2285	2878
	% within row	5.4 %	15.2 %	79.4 %	100.0 %

Table S10. Vaccination Status as a function of Health Status

Contingency Tables

Underlying Health Condition		University Vaccine Mandate Stance			Total
		Neutral	Oppose	Support	
No	Observed	143	399	2077	2619
	% within row	5.5 %	15.2 %	79.3 %	100.0 %
Yes	Observed	12	39	208	259
	% within row	4.6 %	15.1 %	80.3 %	100.0 %
Total	Observed	155	438	2285	2878
	% within row	5.4 %	15.2 %	79.4 %	100.0 %
